# Accuracy of Quick Sequential Organ Failure Assessment Score & Systemic Inflammatory Response Syndrome Criteria in Predicting Adverse Outcomes in Emergency Surgical Patients With Suspected Sepsis: A Prospective Observational Study

**DOI:** 10.7759/cureus.26560

**Published:** 2022-07-04

**Authors:** Amith Sreekanth, Ankit Jain, Souradeep Dutta, Gomathi Shankar, Nagarajan Raj Kumar

**Affiliations:** 1 Surgery, Jawaharlal Institute of Postgraduate Medical Education & Research, Puducherry, IND

**Keywords:** diagnostic test accuracy, sepsis, emergency surgical procedure, sirs, qsofa

## Abstract

Purpose: Due to the mixed population enrolled in different studies i.e., medical and surgical cases, conflicting data exists about the accuracy of quick sequential organ failure assessment (qSOFA) and systemic inflammatory response syndrome (SIRS) scores in predicting adverse outcomes in patients with suspected sepsis presenting to the surgical emergency.

Method: A prospective observational study was done in the department of surgery of a tertiary teaching hospital, India from June 2018 to July 2019. Consecutive patients who visited the surgical emergency department with suspected sepsis were included. Patients were followed up until hospital discharge or death.

Results: Of the 410 patients screened, 287 were included in the analysis. The median age was 52 years (interquartile range, 41 to 61years) and 208 (72.8%) were men. Around 56.8% of patients had intra-abdominal pathology, and 43.2% had skin and soft -tissue infection. Sixty-nine (24%) patients died during their hospitalization, 98 (34.1%) patients had organ dysfunction, and 168 (58.5%) patients needed admission to the intensive care unit (ICU). A higher qSOFA score (≥2) was associated with organ dysfunction, ICU admission, and in-hospital mortality. The specificity, positive predictive value and diagnostic accuracy of qSOFA for organ dysfunction (85.7%, 67.8%, 76.3%), ICU admission (92.4%, 89.3%, 64.5%), and in-hospital mortality (81.6%, 52.4%, 77.4%) was higher than SIRS. The area under the receiver operating characteristic curve for qSOFA for these variables was also higher than for SIRS (0.826 vs. 0.524, 0.823 vs. 0.577, and 0.823 vs. 0.555, respectively).

Conclusion: qSOFA is a better model for predicting adverse outcomes and mortality, organ dysfunction, and ICU admission in surgical patients. However, SIRS indicates intervention requirements in a surgical patient better than qSOFA.

## Introduction

Sepsis is described by Third International Consensus Definitions for Sepsis and Septic Shock (Sepsis-3) Task Force as a critical organ dysfunction due to dysregulated response from the host to the underlying infection [1]. A meta-analysis reported a global incidence of 31.5 million sepsis cases annually; 19.4 million cases among them were severe sepsis [2]. Surgical sepsis accounts for 30% of total cases of sepsis [3].

Commonly used sepsis scoring methods cannot distinguish sterile inflammation due to noninfectious causes from inflammation secondary to infections. Moreover, surgical cases, especially post-operative, have various non-inflammatory causes, leading to a higher score like pain-induced tachycardia and tachypnea and post-operative sedation-induced reduced consciousness [4]. Post-operative cases also display an inflammatory response in the absence of true sepsis.

Systemic inflammatory response syndrome (SIRS) was used to define sepsis in the presence of infection. However, it was abandoned in favor of the quick sequential organ failure assessment (qSOFA) score in patients outside the intensive care unit (ICU) settings in 2016 [1]. Various studies have shown conflicting results about the accuracy of qSOFA when compared with SIRS in predicting adverse outcomes like ICU admission and in-hospital mortality [5,6]. This variation is due to the mixed population enrolled in studies i.e., medical and surgical cases [4,7]. Therefore, this study was done to compare the diagnostic accuracy of qSOFA and SIRS in purely surgical patients with suspected sepsis presenting to the surgical emergency.

## Materials and methods

Data source and ethics review

This prospective observational study was conducted between June 2018 to July 2019 in the Department of Surgery, Jawaharlal Institute of Postgraduate Medical Education and Research, Puducherry, a tertiary care teaching hospital in South India. All adult patients (> 18 years) admitted in the surgical emergency with suspected sepsis were included in the study. Suspected sepsis was defined as patients who were started on antibiotics and/or patients whose blood, pus, or tissue sample has been sent for culture and sensitivity. The patients referred with documented organ failure at admission or with incomplete records were excluded from the study. The study's ethical clearance was obtained from the Institute Ethics Committee (approval number: JIP/IEC/2018/0243). This study is reported in accordance with the Standards for Reporting of Diagnostic Accuracy Studies (STARD) 2015 guidelines [8].

Sample size

The sample size was calculated to be a minimum of 270, considering the area under the curve (AUC) of qSOFA for in-hospital mortality as 0.73 and SIRS as 0.60, the prevalence of 19%, power of 80%, confidence level of 95%, and drop rate of 10% [9]. Taking the AUC of qSOFA for ICU admission to be 0.71 and for SIRS as 0.58, the prevalence of 29.6%, power of 80%, confidence level of 95%, and drop rate of 10%, the minimum sample size was calculated to be 200. Taking the AUC of qSOFA for organ dysfunction to be 0.81 and for SIRS as 0.66, the prevalence of 62.1%, power of 80%, confidence level of 95%, and drop rate of 10% minimum sample size was calculated to be 125. Since the largest of all the three was for in-hospital mortality, 270 was taken as the minimum sample size.

Procedure and outcomes

Case records of all patients on admission were assessed for qSOFA [1] and SIRS [1] scores. A qSOFA or SIRS score of ≥2 was considered positive. Co-morbidities were stratified according to the Charlson co-morbidity index [10]. Case records of all patients were followed up throughout the hospital stay to evaluate the adverse outcomes (organ dysfunction, ICU admission, and in-hospital mortality). Organ dysfunction was defined by an increase in the mSOFA (modified sequential organ failure assessment) score of at least two [11]. An ICU admission > 3 days was defined as a prolonged stay.

Statistical analysis

Data were assessed using Statistical Package for Social Sciences (SPSS) 19 (IBM Corp., Armonk, NY, USA) software. Continuous data were represented as mean ± standard deviation and non-normally distributed variables as median (interquartile range). Categorical variables were expressed as numbers and percentages. Differences were tested for statistical significance using the Chi-square test for categorical variables. The qSOFA and SIRS scores were categorized based on ≥2 cut-off, and diagnostic validity, namely sensitivity, specificity, positive predictive value (PPV), negative predictive value (NPV), and accuracy were calculated for adverse outcomes. The result was summarized as estimates with a 95% confidence interval using the Wilson interval. The receiver operating characteristic (ROC) curve was constructed for all outcomes to ascertain the area under the curve and the significance of the model. A p-value < 0.05 was considered significant for all the statistical tests conducted.

## Results

Study population

**Figure 1:**
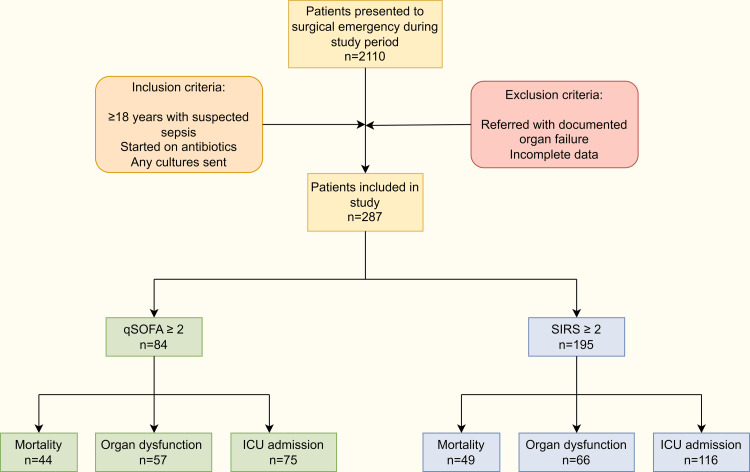
Flow diagram of study ICU: Intensive care unit, qSOFA:  Quick sequential organ failure assessment, SIRS: Systemic inflammatory response syndrome

A total of 410 patients with sepsis were screened. After excluding case records with missing values, 287 patients were included in the study (Figure 1).

The demographic profile and baseline variables are tabulated in Table 1.

**Table 1:** Distribution of baseline characteristics among study patients ICU: Intensive care unit

S.No	Characteristics and Parameters	N=287	%
1	Age (years) - Median age 52 years (interquartile range, 41 to 61years)		
	≤20	14	4.9 %
	21-50	122	42.51%
	51-60	77	26.8%
	>70	74	25.8%
2	Gender		
	Male	209	72.8%
	Female	78	27.2%
3	Charlson index		
	0	70	24.4%
	1-4	164	57.1%
	5-8	50	17.4%
	9	3	1.0%
4	Site of infection		
	Intra-abdominal	163	56.8%
	Skin and soft-tissue infection	124	43.2%
5	Diagnosis		
	Hollow viscus perforation	67	23.3%
	Intestinal obstruction & gangrene	29	10.1%
	Appendicitis and its sequelae	37	12.9%
	Pancreatitis and its sequelae	12	4.2%
	Diabetic foot & Peripheral vascular diseases	50	17.4%
	Cellulitis & Necrotising soft tissue infection	56	19.5%
	Others	36	12.5%
6	Intervention		
	Operative	231	80.5%
	Radiology/Endoscopic guided	16	5.6%
	Conservative	40	13.9%
7	Mortality	69	24%
8	Organ dysfunction	98	34.1%
9	ICU admission	168	58.5%
	≤3days	93	32.4%
	>3days	75	26.1%
7	Cause of death		
	Septic shock	59	20.2%
	Pulmonary embolism	2	0.7%
	Ventilator-associated pneumonia	2	0.7%
	Acute liver failure	2	0.7%
	Cardiogenic shock	4	1.4%

The median age was 52 years (interquartile range, 41 to 61years) with a male:female ratio of 2.7:1. Around 24.4% of patients had a Charlson index of 0, and 81.5% of patients had an index of ≤4. About 56.8% of patients had intra-abdominal etiological pathology, the most common was hollow viscus perforation, and 43.2% of patients had skin and soft-tissue infection. Sixty-nine (24%) patients died during their hospitalization, 98 (34.1%) patients had organ dysfunction, and 168 (58.5%) patients needed ICU admission, out of which 75 patients had a prolonged stay.

SIRS versus qSOFA

The distribution of SIRS and qSOFA scores for different adverse outcomes are shown in Table 2, and Table 3.

**Table 2:** qSOFA & SIRS score for the study population ICU: Intensive care unit, qSOFA:  Quick sequential organ failure assessment, SIRS: Systemic inflammatory response syndrome

Score	Total (287)	Mortality (69)	Organ Dysfunction (98)	ICU admission (168)
qSOFA				
0	120(41.8%)	1(1.4%)	5(5.1%)	28(16.7%)
1	83(28.9%)	24(34.8%)	36(36.7%)	65(38.7%)
2	82(28.6%)	42(60.9%)	55(56.1%)	73(43.5%)
3	2(0.7%)	2(2.9%)	2(2.0%)	2(1.2%)
SIRS				
0	10(3.5%)	0	0	3(1.8%)
1	82(28.6%)	20(29%)	32(32.7%)	49(29.2%)
2	156(54.4%)	35(50.7%)	49(50.0%)	81(48.2%)
3	35(12.2%)	12(17.4%)	15(15.3%)	31(18.5%)
4	4(1.4%)	2(2.9%)	2(2.0%)	4(2.4%)

**Table 3:** Distribution of SIRS and qSOFA scores as per different outcomes ICU: Intensive care unit, qSOFA:  Quick sequential organ failure assessment, SIRS: Systemic inflammatory response syndrome

S.no	Outcomes		SIRS			qSOFA		
			Negative (<2) (N=92)	Positive (≥2) (n=195)	p-Value	Negative (<2) (N=203)	Positive (≥2) (n=84)	p-Value
1	Mortality	Yes (n=69)	20(29%)	49(71%)	0.531	25(36.2%)	44(63.8%)	<0.001
		No (n=218)	72(33%)	146(67%)		178(81.7%)	40(18.3%)	
2	Organ dysfunction	Yes (n=98)	32(32.7%)	66(67.3%)	0.876	41(41.8%)	57(58.2%)	<0.001
		No (n=189)	60 (31.7%)	129(68.3%)		162(85.7%)	27(14.3%)	
3	Intervention required (Operative and Endoscopic/Radiologic guide)	Yes (n=247)	77(31.2%)	170(68.8%)	0.426	169(68.4%)	78(31.6%)	0 .032
		No (n=40)	15(37.5%)	25(62.5%)		34(85.0%)	6(15.0%)	
4	ICU admission	Yes (n=168)	52(31%)	116(69%)	0.634	93(55.4%)	75(44.6%)	<0.001
		No (n=119)	40(33.6%)	79(66.4)		110(92.4%)	9(7.6%)	
5	ICU duration (days)		(N=52)	(n=116)		(N=93)	(n=75)	
		≤ 3days (n=93)	35(37.6%)	58(62.4%)	0.037	52(55.9%)	41(44.1%)	0.872
		>3days (n=75)	17(22.7%)	58(77.3%)		41(54.7%)	34(45.3%)	

Contrary to the SIRS score, a higher qSOFA score was associated with organ dysfunction, ICU admission, intervention required, and in-hospital mortality. However, higher SIRS scores were seen to be associated with prolonged ICU stay (p=0.037), unlike the qSOFA score (p=0.872).

The sensitivity, specificity, PPV, NPV, and diagnostic accuracy of qSOFA and SIRS are tabulated in Table 4.

**Table 4:** Diagnostic parameters of qSOFA & SIRS score for different outcomes Values in parentheses represent 95% confidence intervals for predictive values. AUC: Area under the curve, ICU: Intensive care unit, qSOFA:  Quick sequential organ failure assessment, NLR: Negative likelihood ratio, NPV: Negative predictive value, PLR: Positive likelihood ratio, PPV: Positive predictive value, SIRS: Systemic inflammatory response syndrome

Characteristics	Mortality		Organ Dysfunction		ICU Admission		Intervention	
	qSOFA	SIRS	qSOFA	SIRS	qSOFA	SIRS	qSOFA	SIRS
Sensitivity(%)	63.8 (51.3-75)	71.0 (58.8-81.3)	58.2 (47.8-68.1)	67.4 (57.1-76.5)	44.6 (37.0-52.5)	69.1 (61.5-75.9)	31.6 (25.8-37.8)	68.8 (62.7-74.6)
Specificity(%)	81.6 (75.9-86.6)	33.0 (26.8-39.7)	85.7 (79.9-90.4)	31.8 (25.2-38.9)	92.4 (86.1-96.5)	33.6 (25.2-42.9)	85.0 (70.2-94.3)	37.5 (22.7-54.2)
PLR	3.5 (0.3-0.6)	1.06 (0.9-1.3)	4.1 (2.8-6)	0.99 (0.8-1.2)	5.9 (3.1-11.3)	1.04 (0.9-1.2)	2.1 (0.98-4.5)	1.1 (0.9-1.4)
NLR	0.4 (2.5-4.9)	0.9 (0.6-1.3)	0.5 (0.4-0.6)	1.03 (0.72-1.46)	0.6 (0.5-0.7)	0.9 (0.7-1.3)	0.8 (0.7-0.9)	0.8 (0.5-1.3)
PPV(%)	52.4 (44.1-60.5)	25.1 (21.9-28.6)	67.8 (58.9-75.7)	33.8 (30.2-37.7)	89.3 (81.3-94.1)	59.5 (55.5-63.3)	92.9 (85.9-96.5)	87.2 (84.1-89.8)
NPV(%)	87.7 (83.8-90.7)	78.3 (70.4-84.5)	79.8 (75.6-83.4)	65.2 (56.9-72.7)	54.2 (50.6-57.8)	43.5 (35.4-51.9)	16.8 (14.7-19.0)	16.3 (11.1-23.2)
Diagnostic accuracy (%)	77.4 (72-82.1)	42.2 (36.4-48.1)	76.3 (71.0-81.1)	43.9 (38.1-49.8)	64.5 (58.6-70.0)	54.4 (48.4-60.2)	39.0 (33.4-44.9)	64.5 (58.6-70.0)
AUC	0.823	0.555	0.826	0.524	0.823	0.577	0.675	0.558

**Figure 2:**
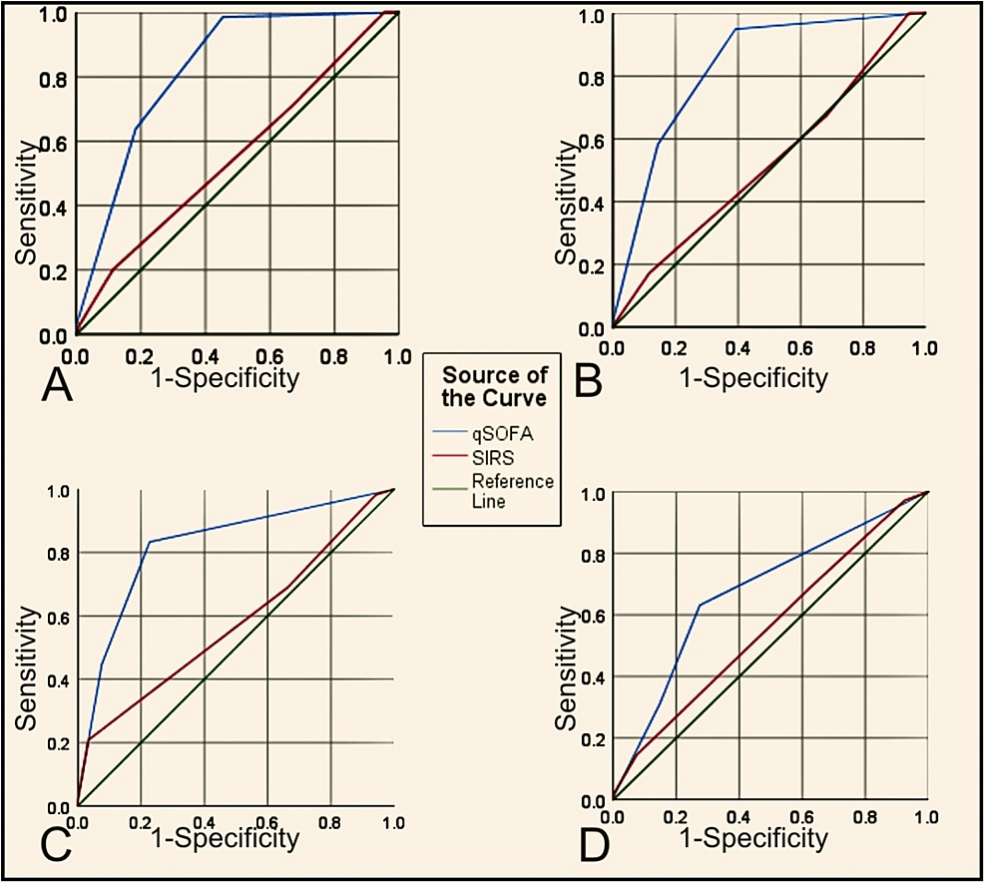
ROC curve comparing qSOFA and SIRS. (A) Mortality; (B) Organ dysfunction; (C) ICU admission; (D) Intervention required qSOFA:  Quick sequential organ failure assessment, ROC: Receiver operating characteristic curve, SIRS: Systemic inflammatory response syndrome

**Figure 3:**
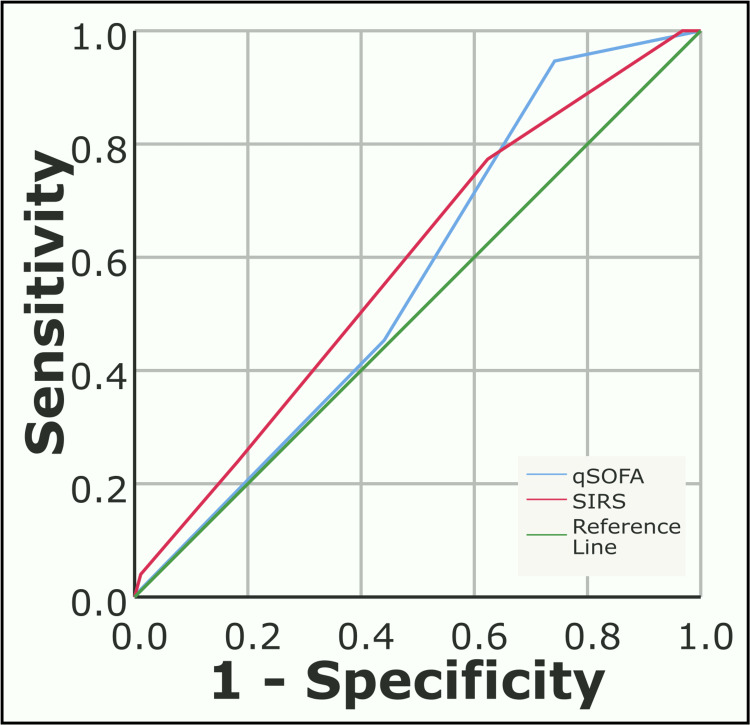
ROC curve comparing qSOFA and SIRS for ICU stay >3 days ICU: Intensive care unit, qSOFA:  Quick sequential organ failure assessment, ROC: Receiver operating characteristic curve, SIRS: Systemic inflammatory response syndrome

The ROC curve was constructed to compare the predictive ability of qSOFA and SIRS (Figure 2, Figure 3).

The specificity, PPV and diagnostic accuracy of qSOFA for organ dysfunction (85.7%, 67.8%, 76.3%), ICU admission (92.4%, 89.3%, 64.5%), and in-hospital mortality (81.6%, 52.4%, 77.4%) was higher than SIRS. The area under the receiver operating characteristic curve (AUROC) for qSOFA for these variables was also higher than for SIRS, suggesting a better prediction model. Therefore, qSOFA is a better model for predicting adverse outcomes in surgical patients with sepsis. The ROC curve of two scores for predicting prolonged ICU stay shows poor prediction ability.

Comparing the predictive ability of two scores for intervention requirements, SIRS had a lower specificity, similar NPV and PPV, and higher sensitivity and diagnostic accuracy. Therefore, SIRS predicts intervention requirements in a surgical patient better than qSOFA.

## Discussion

Sepsis was defined by two International consensus conferences in 1991 And 2001 as ≥2 SIRS criteria with a confirmed or suspected infection. However, SIRS was found to have poor discrimination ability for infective vs. sterile inflammations like pancreatitis, myocardial infarction, and burns [12]. Also, a retrospective study showed poor specificity of SIRS as it missed one in eight patients with infection and organ dysfunction [13]. Therefore, Sepsis-3 2016 abandoned SIRS and introduced the qSOFA score to identify septic patients outside ICU settings [1]. Though qSOFA and SIRS have been tested and validated in multiple settings worldwide, ICU and non-ICU, prospective [6,14,15], and retrospective [8,16,17], they have not been studied earlier in the context of only acute surgical patients in an emergency setting. Although many studies have compared qSOFA and SIRS in the surgical population they only included post-operative cases already admitted in cardiothoracic [18,19] or surgical ICUs [20]. The present study prospectively followed 287 patients with suspected sepsis admitted to the surgical emergency department. A higher qSOFA score was associated with organ dysfunction, ICU admission, and in-hospital mortality. Therefore, qSOFA is a better model for predicting surgical patients' adverse outcomes due to its higher specificity and diagnostic accuracy, and slightly low sensitivity. However, SIRS indicates intervention requirements in a surgical patient better than qSOFA.

The most common diagnosis in the present study was hollow viscus perforation (23.3%), followed by necrotizing soft-tissue infection and diabetic foot. Respiratory infection or febrile infections has been the most common etiology in most studies suggesting a mixed population, the majority being medical patients [6,15,17,21,22]. Overall in-hospital mortality in the present study was 24% (69 patients). Many studies have reported mortality in the range of 5% to 20% [5,15,23]. The present study was done in a tertiary care center with most of the patients referred from other hospitals. As a result, most cases present in an advanced stage of sepsis. Moreover, there may be a delay in initiating treatment at primary health care, thus increasing mortality.

Our study's AUROC for mortality for qSOFA score was similar to the values seen in Sepsis-3 criteria studies conducted in non-ICU settings (0.81) [1,24]. The AUROC of qSOFA has been higher than SIRS for mortality, organ dysfunction, and ICU admission in various other studies [5,9,15,25]. However, Song et al., in their metanalysis, reported higher AUROC of SIRS for ICU admission and similar AUROC for mortality [12]. In our study, the AUROC of qSOFA was found to be higher than SIRS across all the categories of mortality, organ dysfunction, ICU admission, and requirement of intervention. This could be due to purely surgical patients included in the present study. Due to the differences in study patients, the results of different studies may not be comparable.

The qSOFA has a strong association with mortality and organ dysfunction with higher specificity and lower sensitivity than SIRS [9,12,15,17,21,25,26]. Therefore, qSOFA could miss cases with sepsis leading to life-threatening complications, whereas a higher sensitivity of SIRS would lead to overdiagnosis and wastage of resources. Some authors [15,25] have preferred specificity over sensitivity for a suitable sepsis scoring system, whereas authors such as Askim et al. [14] have preferred sensitivity. Many options are suggested to increase the sensitivity of qSOFA. Firstly, the worst qSOFA score during the entire stay of the patient can be used [12,15]. Second, additional clinical factors such as age, nursing home residence, arterial pH, lactate, and end-tidal carbon dioxide concentrations can be used [27-29]. However, contradictory evidence about the usefulness of lactate is mentioned in the literature [1,15,24]. Third, using a cut-off of ≥ 1 for the qSOFA score can increase sensitivity but at the cost of specificity [9,17,30]. In the present study, the sensitivity of qSOFA in predicting adverse outcomes was highest at a cut-off ≥1. A qSOFA score of 3 had the highest specificity but with the least sensitivity compared to the SIRS score. Some authors have suggested differential cut-off as per need; a qSOFA ≥1 can be used as a better screening tool, whereas qSOFA ≥2 can be used as a cut-off to identify high-risk cases, thereby properly utilizing resources [9,17]. Jiang et al. recommended SIRS as a tool for early identification of cases and qSOFA to identify high-risk cases, and that both scoring systems be used depending on the health care setting i.e., qSOFA in places with less sepsis-related mortality and SIRS in resource-rich settings with high sepsis-related mortality [26]. A study by Serafim et al. suggested using both the scores in a two-step process, i.e., using a more sensitive score like SIRS to screen patients and among those positive for SIRS, apply qSOFA for the proper utilization of resources [30].

Early recognition of sepsis and prompt aggressive fluid resuscitation and administration of antimicrobials are crucial to improving outcomes and decreasing sepsis-related mortality [1,12]. The qSOFA score has an advantage as a simple bedside tool with few variables, no necessary laboratory results, and ease of repeated assessment over time if the patient’s condition worsens. Due to non-dependence on investigation such as leukocytosis or blood lactate, qSOFA can detect sepsis patients early, immediately upon arrival [15]. The qSOFA, owing to its higher specificity, would be superior to the SIRS score in a developing nation because of resource-poor settings such as lack of ICU beds, staff, and infrastructure, especially in most primary health care setups [1]. Therefore, using the qSOFA score, one can utilize the available resources appropriately. However, qSOFA criteria should be used only as a guiding tool to investigate organ dysfunction further, initiate or escalate treatment as appropriate, or admission to ICU care if such actions have not already been undertaken. Failure to meet qSOFA criteria should not lead to a deferral of investigation or delay in treatment as deemed necessary by the treating health care personnel [1].

Strengths of the study

Ours was a prospective study with a larger sample size. As per our knowledge, no other study has prospectively compared the diagnostic accuracy of both sepsis scores in predicting mortality or ICU admission in a surgical emergency setting. Most of the studies conducted previously are in a purely medical setup or a mixed population. The studies with purely surgical patients had either post-operative cases only or cases already admitted to ICU.

Limitations of the study

Ours was a single-center study. We have used the modified SOFA score to identify organ dysfunction. This might have led to variation in the prediction of organ dysfunction. The scores were calculated only at admission, and the qSOFA score trend or worst qSOFA score was not considered. Outcomes of only admitted cases and only in-hospital mortality were studied. The present study did not follow up with patients after discharge who may have been readmitted or died.

## Conclusions

This study was done to compare the diagnostic accuracy of qSOFA versus SIRS in predicting the adverse outcomes in patients with suspected sepsis presenting to the surgical emergency and it was found that qSOFA is a better model for predicting the adverse outcomes in terms of mortality, organ dysfunction, and ICU admission. However, SIRS predicts intervention requirements in a surgical patient better than qSOFA. The sensitivity of qSOFA score decreases and specificity increases as the cut off value increases from ≥1 to ≥3.
